# Thromboembolic disease in testicular germ cell tumors—real-world evidence of three Portuguese institutions

**DOI:** 10.3389/fonc.2026.1772608

**Published:** 2026-03-02

**Authors:** Joana Albuquerque, Martim Oliveira, Diana Neto-Silva, Inês Margarido, Jorge Correia, Carlota Baptista, Madalena Machete, Rita Bizarro, João Rato, João Godinho, José Alberto Teixeira, José Luís Passos-Coelho

**Affiliations:** 1 Oncology Department of Hospital da Luz, Lisbon, Portugal; 2 Católica University of Medicine, Universidade Católica Portuguesa, Lisbon, Portugal; 3 Oncology Department, Hospital da Luz, Setúbal, Portugal; 4 Oncology Department of Unidade Local de Saúde Loures-Odivelas, Loures, Portugal

**Keywords:** central venous catheter, cisplatin, risk assessment, testicular germ cell cancer, thromboembolic disease, thromboprophylaxis

## Abstract

**Introduction:**

Testicular germ cell tumors (TGCTs) are highly curable malignancies with long-term overall survival (OS) exceeding 90%. Thromboembolic (TE) events are a relevant treatment-related complication, reported in approximately 10% of patients. This study aimed to evaluate the incidence, risk factors, and prognostic impact of TE in TGCT.

**Methods:**

We performed a retrospective multicenter cohort study including 136 post-pubertal male patients with histologically confirmed TGCT treated between 2007 and 2021 at three Portuguese centers. The primary endpoint was to characterize the population of TGCT patients with TE. Secondary endpoints included TE incidence and its impact on OS and progression-free survival (PFS). Identification of clinical, pathological, and treatment-related factors associated with increased TE risk was an exploratory endpoint.

**Results:**

Seven patients (5.1%) developed a TE event, all in advanced/recurrent disease (14.6% in this subgroup). No TE occurred in stage I patients, including those treated with adjuvant chemotherapy. Visceral metastases (pulmonary and extra-pulmonary) and poor IGCCCG prognosis were associated with TE (*p* < 0.05). All TE patients had a central venous catheter (CVC), although only two had catheter-related thrombosis (*p* = 0.019). For advanced stages, survival outcomes did not differ significantly, with 5-year OS of 71.4% vs. 86.2% (*p* = 0.22) and PFS of 47.6% vs. 75.5% (*p* = 0.23) in TE versus non-TE groups, respectively. Most events (86%) occurred within 30 days of chemotherapy initiation, with pulmonary embolism as the most frequent presentation. Neither the Khorana nor the ONKOTEV scores predicted TE.

**Discussion:**

TE in TGCT occurred only in patients with advanced disease, was linked to tumor burden and CVC use, and was not predicted by current models. These findings highlight the need for TGCT-specific risk tools and prospective studies on risk-adapted prophylaxis.

## 1 Introduction

Testicular germ cell tumors (TGCT) are the most frequent solid malignancies in young adult male patients, typically affecting individuals between 15 and 40 years. Although they account for only 1% of all male cancers, their incidence has been steadily rising in Western countries over the past decades (1, 2). TGCTs are highly curable, with long-term survival exceeding 95% in the early stage of the disease and approaching 90% at 10 years, even in advanced stages, largely due to the effectiveness of cisplatin-based chemotherapy (1, 3, 4). Given the young age of the affected population and the high curability of the disease, optimizing supportive care and minimizing treatment-related morbidity has become increasingly important to preserve long-term health and quality of life (5).

Among the potential complications of treatment, thromboembolism (TE)—encompassing deep vein thrombosis (DVT), pulmonary embolism (PE), and other thrombotic events—has emerged as a clinically relevant and increasingly recognized concern in patients with TGCT undergoing systemic therapy. The incidence of TE is approximately 10% in patients with advanced TGCT, occurring mostly during chemotherapy treatment, which is notably high compared to other solid tumors, particularly accounting for the younger age and low comorbidities of these patients (6, 7). Major thrombotic risk factors include bulky retroperitoneal lymph nodes, elevated tumor markers after orchiectomy, advanced or poor-risk disease according to the International Germ Cell Cancer Collaborative Group (IGCCCG) classification, use of central venous catheters (CVC), and the thrombogenic effects of cisplatin-based chemotherapy (7–9).

The biological mechanisms underlying cancer-associated thrombosis are multifactorial and include tumor-related hypercoagulability, endothelial dysfunction, chemotherapy-induced vascular injury, and pro-inflammatory cytokine release (10, 11). In TGCT specifically, bulky retroperitoneal lymph nodes may cause mechanical compression of major vessels, further predisposing patients to thrombosis, while cisplatin independently increases the risk of both arterial and venous events through its pro-thrombotic effect driven by several distinct biological mechanisms (7, 8, 12).

Firstly, cisplatin exerts direct toxic effects on the vascular endothelium, leading to endothelial cell apoptosis and the subsequent exposure of the subendothelial matrix (13), which triggers the release of pro-coagulant ultra-large von Willebrand factor multimers and activates the extrinsic coagulation pathway. Moreover, cisplatin therapy has been associated with the induction of pro-inflammatory cytokines and the generation of microparticles that further amplify thrombin production and platelet activation (14, 15). Understanding these mechanisms is crucial, as the peak of this vascular damage often occurs early during the first cycles of treatment, coinciding with the highest risk period for clinical thromboembolic events (6).

Risk prediction in this setting remains challenging. While several predictive models, such as the Khorana Score and OnkoTEV Score, have been developed to estimate the risk of TE in cancer patients receiving chemotherapy, their performance in TGCT populations remains uncertain. These models typically incorporate variables such as tumor site, blood counts, body mass index, and other clinical parameters, yet they may lack sensitivity and specificity in younger patients with highly chemosensitive malignancies (16, 17). Furthermore, pharmacological thromboprophylaxis in high-risk populations has been evaluated in small or non-randomized studies, yielding conflicting results on efficacy and bleeding risk (6, 18). The most recent ESMO-EURACAN guidelines recommend considering thromboprophylaxis with low molecular weight heparin (LMWH) in patients with advanced TGCT under chemotherapy (excluding those with central nervous system and/or hemorrhagic lesions), especially for those who have a Khorana score of 3 or more, retroperitoneal lymph nodes that are more than 3.5 cm, stage III TCGT or IGCCCG poor prognosis group (level of evidence: IIA). Peripheral venous access should be used instead of an indwelling vascular access device (19, 20).

The clinical consequences of TE should not be neglected, as it can delay surgical interventions or chemotherapy in a highly curable disease; mobility and pain may negatively impact the quality of life (22), and it can also be a life-threatening event, being one of the leading causes of noncancer deaths among cancer patients (6, 9). Two recent large studies reported a five- to sevenfold increased risk of death from cardiovascular disease, including TE, during the first year after cisplatin-based ChT (21, 22). Data from the GCT Spanish Group point to a significant reduction in progression-free survival (PFS) (HR 2.29, *p* = 0.02) and OS (HR 5.14, *p* <.001) once TE has been documented (23).

Despite these findings, prospective randomized studies and real-world data specific to TGCT patients are scarce. In this study, we report the incidence and clinical characteristics of TE and explore its impact on survival outcomes. A better understanding of TE risk factors, including tumor burden, treatment intensity, and validated clinical scores such as the Khorana and OnkoTEV, may enhance individual risk stratification. These insights could guide the selective use of prophylactic anticoagulation, particularly in high-risk patients, and contribute to improving both oncologic and supportive care strategies in this unique and highly curable population.

## 2 Materials and methods

### 2.1 Study design and patient population

We performed a multicenter, retrospective, observational cohort study conducted across three Portuguese institutions. Eligible patients were identified through institutional databases and electronic medical records.

The study population comprised post-pubertal male patients with histologically confirmed TGCT, including both seminomatous and non-seminomatous subtypes, diagnosed between January 1, 2007 and December 31, 2021. The inclusion criteria required treatment at one of the participating centers and the availability of at least one documented follow-up visit related to disease management or surveillance.

Patients were excluded if they had a malignant histological diagnosis other than TGCT, were pre-pubertal at the time of diagnosis, or had a concurrent second primary malignancy under active investigation or treatment at the time of TGCT diagnosis, except for localized non-melanoma skin cancers (i.e., basal cell or squamous cell carcinoma). Additionally, patients seen exclusively for second-opinion consultations, without subsequent clinical follow-up recorded within the participating institutions, were excluded from the analysis.

### 2.2 Endpoints

The primary endpoint of this study was to describe the clinical, pathological, and demographic characteristics of the TGCT population with a TE.

The secondary endpoint included the following:

- To assess the impact of TE on OS in patients with TGCT
- To assess PFS for advanced/recurrent disease and disease-free survival (DFS) for early stage, according to the occurrence of TE
- To determine the incidence of TE events in this patient population
- To characterize the clinical presentation (symptomatic or asymptomatic), type of TE (arterial or venous), for venous TE events, anatomical location (e.g., pulmonary embolism, deep vein thrombosis or inferior vena cava thrombosis), and timing of TE events relative to the chemotherapy treatment timeline (30 days before, during chemotherapy, or up to 90 days after completion of chemotherapy).
- To evaluate TE-related 30-day mortality
- Exploratory endpoint aimed to identify clinical, pathological, and treatment-related factors associated with an increased risk of TE, including the following:
- Tumor-related factors and disease burden: Histologic subtype (seminoma or non-seminoma/mixed), stage according to the American Joint Committee on Cancer (AJCC) 8th edition—including site of metastases (pulmonary, non-pulmonary visceral, and lymph node metastases) and IGCCCG risk group for advanced disease (favorable, intermediate, and poor risk)
- Patient characteristics: age, smoking history, Eastern Cooperative Oncology Group Performance Status (ECOG-PS), and TE risk scores (Khorana Score and ONKOTEV Score).
- Treatment-related variables: regimen and number of chemotherapy cycles, response to treatment, total number of treatment lines for advanced disease, CVC use, clinical context of TE detection (inpatient vs. outpatient).

TE was defined as any radiologically confirmed thrombotic event involving the deep venous system (DVT), lung vessels (PE), inferior vena cava (IVC), or other vascular structures, occurring from diagnosis until last follow-up or death. Patients considered at high risk for TE were those treated with cisplatin-based chemotherapy for advanced disease and/or with elevated Khorana (≥3) or ONKOTEV (≥4) scores. Primary thromboprophylaxis was defined as any pharmacologic anticoagulation initiated before or during chemotherapy, following multidisciplinary tumor board discussion and an individualized assessment of thrombosis and bleeding risk.

The 30-day mortality due to TE included deaths occurring within 30 days of a documented TE event, in which TE was a direct or contributing cause.

Survival outcomes were defined as follows: for stage I disease, DFS was measured from the date of diagnosis to the first documented event, defined as recurrence, second primary cancer, or death from any cause, whichever occurred first. For advanced or recurrent disease, PFS was measured from the date of diagnosis of advanced or recurrent disease to the date of objective disease progression or death from any cause, whichever occurred first. Overall survival (OS) was defined as the time from initial TGCT diagnosis to death from any cause. Patients without an event were censored at the date of last follow-up.

Treatment efficacy was evaluated by serum tumor markers and imaging exams per the treating physician’s discretion and reviewed by institutional radiologists. Tumor response was categorized according to RECIST v1.1 criteria as complete response (CR), partial response (PR), stable disease (SD), or progression disease (PD). The overall response rate (ORR) was defined as the proportion of patients achieving either CR or PR.

Serum levels of AFP, β-hCG, and LDH were interpreted using local laboratory reference values for categorization as normal or elevated.

Finally, hospitalization rate due to TE was defined as the proportion of patients requiring inpatient care for TE-related events from the date of TE diagnosis up to 180 days after chemotherapy completion.

### 2.3 Data collection, cleaning, and management

Data were retrospectively extracted from the electronic medical records of three participating Portuguese institutions. Follow-up information for all included patients was collected up to December 31, 2023.

A rigorous and systematic variable selection process was undertaken, categorized as follows:

- Thromboembolic events, including clinical context of TE detection, timing of event relative to chemotherapy (before, during, or until 90 days after completion of chemotherapy), type (arterial, venous, or mixed), anatomical location (e.g., pulmonary, extremities, inferior vena cava), and clinical presentation (symptomatic or asymptomatic).
- Survival outcomes, specifically DFS, PFS, and OS.
- Tumor characteristics, including histologic subtype, staging according to the AJCC 8th edition, IGCCCG risk group (for advanced/recurrent disease), and primary tumor size, presence of lymphovascular invasion, rete testis invasion, teratoma and/or embryonal cell carcinoma component (for stage I disease, according to risk factors defined for seminoma and non-seminoma tumors).
- Treatment-related data, comprising the treatment setting (adjuvant or advanced stage), regimen, number of chemotherapy cycles, response to treatment according to RECIST v 1.1 criteria, and CVC use.
- TE risk stratification scores, including the Khorana score—incorporating platelet count ≥350 × ^9^/L, hemoglobin <10 g/dL or use of erythropoiesis-stimulating agents, leukocyte count >11 × ^9^/L, and BMI ≥35 kg/m²—and the ONKOTEV score, which includes the Khorana score components along with metastatic disease, vascular/lymphatic compression, and prior history of TE.
- Patient characteristics, including age, ECOG PS, smoking history, and history of anticoagulation therapy before TE.

Missing data were managed according to clinical relevance and the proportion of missingness. Outliers in continuous variables, particularly LDH, AFP, and β-hCG, were identified via descriptive statistics and retained when consistent with known patterns of advanced-stage disease or unfavorable prognostic profiles.

The final cleaned and recoded dataset was exported to IBM SPSS Statistics^®^ software version 30.0 for statistical analysis.

### 2.4 Statistical analysis

All analyses were conducted using IBM SPSS^®^ Statistics software (version 30.0). Descriptive statistics were used to summarize baseline demographic, clinical, tumor, and treatment characteristics, along with incidence and characteristics of TE events. TE-related 30-day mortality was reported as the proportion of deaths occurring within 30 days of the thromboembolic event.

The influence of the collected variables on the TE occurrence was assessed with binomial logistic regression. TE occurrence was the dependent variable. Independent variables were previously described as factors that could influence TE occurrence, such as stage according to AJCC 8th edition, site of metastases, prognosis group per IGCCCG classification, CVC use, Khorana and ONKOTEV scores, and number of chemotherapy lines for advanced disease. A top-down approach (univariate *P* < 0.11) was used to obtain the final model. Multivariable logistic regression was not performed due to the small number of events and the risk of model overfitting.

Survival analyses were similarly limited to univariate approaches, given the small number of observed events. The association between individual variables and DFS, PFS, or OS was evaluated using univariate Cox proportional hazards regression. Kaplan–Meier survival curves were generated for selected categorical variables, and group comparisons were performed using the log-rank test.

Missing data and outliers were handled according to predefined protocols. All statistical tests were two-sided, with ***p <***0.05 considered statistically significant.

### 2.5 Ethical considerations

The study was approved by the Institutional Review Board/Independent Ethics Committee (IRB/IEC) of participating centers and designed according to Good Clinical Practice guidelines and the Declaration of Helsinki. Waiver of informed consent was requested by the investigators and accepted by the IRB/IEC owing to the retrospective and non-interventional nature of the study. Clinical data were treated with pseudonymization and kept accessible only to the investigators.

## 3 Results

**Figure 1:**
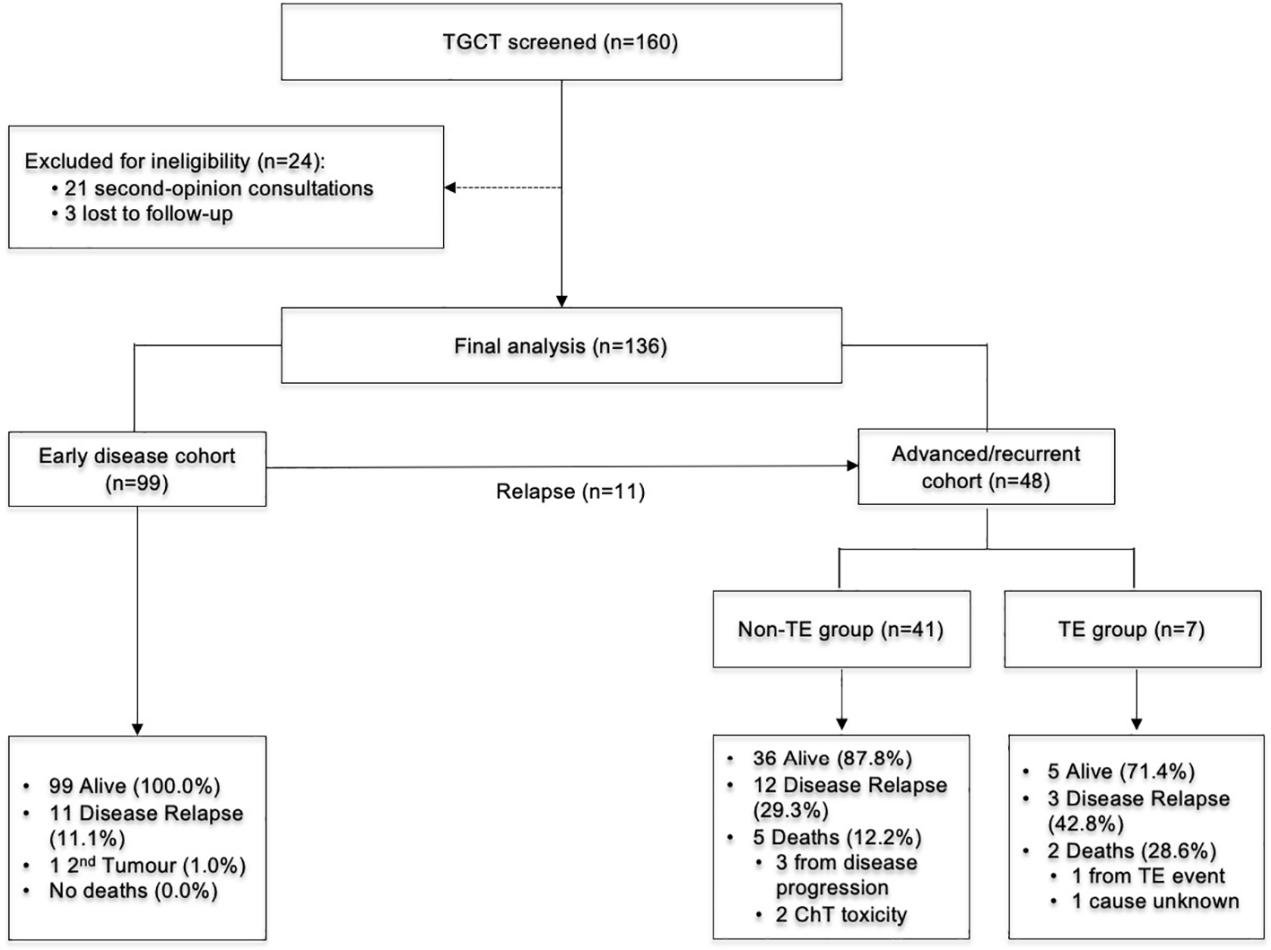
CONSORT diagram. ChT, Chemotherapy; TE, Thromboembolism; TGCT-Testicular Germ Cell Tumor.

Between January 2007 and December 2021, 160 patients with TGCT were screened; 136 met all of the eligibility criteria and constituted the study population. Seven of these 136 patients (5.1%) experienced a TE event during follow−up, and all events occurred in the subgroup with advanced or recurrent disease (**Figure 1**).

For subsequent analyses, the patients were divided into two clinical cohorts:

- Early−stage cohort: stage I disease managed with orchiectomy ± adjuvant therapy
- Advanced/recurrent cohort: stages II–III disease at initial presentation or recurrence after prior stage I management

### 3.1 Early stage cohort (stage I)

Of the 136 patients included, 99 (72.8%) had stage I disease [72 (72.7%) stage IA and 27 (27.3%) stage IB], with a median age of 35.0 years (range, 20–61). No TE events were recorded in this cohort, and all patients evaluated by using Khorana and ONKOTEV scores were classified as low risk.

Seminoma was present in 59 patients (59.6%). Of these, 15 patients (25.4%) had tumors ≥ 40 mm, 11 (18.6%) had rete testis invasion, and six (10.2%) had both risk factors for recurrence. Based on these risk factors, 16 patients received adjuvant treatment with one cycle of carboplatin (AUC 7). Non-seminoma/mixed tumors were diagnosed in 40 patients (40.4%), of whom 18 (45%) had an embryonal cell carcinoma component, one (2.5%) presented with lymphovascular invasion, and three (7.5%) had the two risk factors. Adjuvant BEP (bleomycin, etoposide, and cisplatin) was prescribed to six patients according to the risk of recurrence.

With a median follow-up of 58.2 months (95% CI, 49.0–67.5), 11 patients had recurrence of disease (11.1%) and one (1.0%) had a second tumor; the 5-year DFS was 87.7% (95% CI, 84.1%–93.1%). No deaths were recorded, and five patients were lost to follow-up. Detailed clinicopathological characteristics, stratified by histology and specific risk factors, are summarized in **Table 1**.

**Table 1:** Demographic and clinical characteristics of patients with stage I TGCT.

Variable	Stage I TGCT population (n=99)
**Age (median, range)**	35.0 (20-61)
**Smoking history (n, %)**	
Yes	14 (14.1)
No	85 (85.9)
**ECOG-PS = 0 (baseline) (n, %)**	99 (100)
Stage at diagnosis*(n, %)	
IA	72 (72.7)
IB	27 (27.3)
**Seminoma (n, %)**	**59 (59.6)**
Risk factor	
Tumors ≥ 40mm (only)	15 (25.4)
*Rete testis* invasion (only)	11 (18.6)
Both	6 (10.2)
None	27 (45.8)
Adjuvant Carboplatin AUC 7	**16**
Tumors ≥ 40mm (only)	6
*Rete testis* invasion (only)	4
Both risk factors	6
None risk factor	0
**Non-Seminoma/mixed tumours (n, %)**	**40 (40.4)**
Risk factor	
Embryonal cell carcinoma	18 (45.0)
Lymphovascular invasion	1 (2.5)
Both	3 (7.5)
Adjuvant BEP	**6**
Embryonal cell carcinoma	2
Lymphovascular invasion	0
Both risk factors	3
None risk factor	1
No. BEP cycles (median, range)	2 (1-2)
**TE events**	**0 (0)**
Khorana Score ^†^	
Low risk	31 (100)
High risk	0 (0)
ONKOTEV Score ^†^	
Low risk	31 (100)
High risk	0 (0)

* Staging according to the American Joint Committee on Cancer (AJCC) 8th edition

^†^ Only available for 31 patients.

BEP, Bleomycin, Etoposide and Cisplatin; ChT; TE, Thromboembolism; TGCT, Testicular Germ Cell Tumor.

### 3.2 Advanced stage/recurrent cohort

A total of 48 patients, with a median age of 36.0 years (range, 18–56) and ECOG PS of 0, were treated for recurrent or advanced TGCT: 11 (22.9%) had progressed after treatment for stage I TGCT (all on surveillance without adjuvant therapy) and 37 (77.1%) presented with advanced disease at diagnosis. According to IGCCCG criteria, 32 patients (66.7%) were good risk, one (2.1%) intermediate, and 10 (20.8%) poor risk. Histology included 28 non-seminomas (58.3%) and 20 seminomas (41.7%), with metastatic disease primarily on lymph nodes (n = 33, 68.8%), followed by non-pulmonary visceral (n = 8, 16.7%) and lung involvement (n = 5, 10.4%).

BEP was the first-line treatment regimen (n = 40; 83.3%), with an overall response rate (ORR) of 91.3% (85.0% for non-seminomatous/mixed tumors and 100% for seminoma). Treatment intensity varied according to the risk: good-risk patients received a median of three cycles (range, 1–4), while intermediate/poor-risk patients received four (range, 0–4). Three patients died before or during first-line therapy. The median number of treatment lines was one (range, 0–3), with five patients receiving second-line TIP (paclitaxel, ifosfamide, and cisplatin), and one patient had third-line high-dose chemotherapy (HDCT), with ICE (fosfamide carboplatin plus etoposide) and autologous stem cell transplant (ASCT).

A CVC was placed in 27 patients (56.3%), including all those requiring multiple treatment lines. Khorana (KS) and ONKOTEV scores (available for 42 patients) were predominantly low, with only three (6.3%) and six (14.6%) patients classified as high risk (KS ≥3 and ONKOTEV≥2), respectively. No patient had received venous thromboembolism prophylaxis prior to the TE. Detailed baseline characteristics and risk stratification for this cohort are summarized in **Table 2**.

**Table 2:** Demographic and clinical characteristics of patients with advanced or recurrent/relapse TGCT.

Variable	Overall population (n=48) (n, %)	Non-TE group (n=41) (n,%)	TE-group (n=7) (n,%)	p-value*
**Smoking history**				0.877
Yes	6 (13.1)	5 (12.8)	1 (14.3)	
No	40 (86.9)	34 (87.2)	6 (85.7)	
**Site of metastases ^†^**				0.021
LN (only)	33 (66.7)	31 (75.6)	2 (28.5)	0.174
Lung (w/ or wo/ LN)	5 (10.4)	4 (9.8)	1 (14.3)	0.020
Visceral non-lung (w/ or without lung/LN)	8 (16.7)	4 (9.8)	4 (57.1)	0.005
**Stage at diagnosis ^‡^**				0.074
I	11 (22.9)	11 (26.8)	0 (0.0)	
II	20 (41.7)	18 (43.9)	2 (28.6)	
III	17 (35.4)	12 (29.3)	5 (71.4)	
**Histology**				0.447
Seminoma	20 (41.7)	18 (43.9)	2 (28.6)	
Non-seminoma	28 (58.3)	23 (56.1)	5 (71.4)	
**IGCCCG risk group**				0.003
Good	32 (66.7)	30 (73.2)	2 (28.6)	
Intermediate	1 (2.1)	0 (0.0)	1 (14.3)	
Poor	10 (20.8)	6 (14.6)	4 (57.1)	
*Unknown*	5 (10.4)	5 (12.2)	0 (0.0)	
**1^st^ line treatment**				0.372
BEP/EP	41 (84.8)	35 (85.4)	6 (85.7)	
Other regimens ^§^	2 (8.7)	1 (2.4)	1 (14.3)	
No treatment ^¶^	1 (4.3)	1 (2.4)	0 (0.0)	
*Missing*	4 (2.2)	4 (9.6)	0 (0.0)	
**Response to treatment**				0.526
CR	12 (25.0)	11 (26.8)	1 (14.3)	
PR	21 (43.8)	17 (41.4)	4 (57.1)	
SD	0 (0.0)	0 (0.0)	0 (0.0)	
PD	3 (6.3)	2 (4.9)	1 (14.1)	
*Not available*	12 (25.0)	11 (26.8)	1 (14.1)	
**No. cycles of ChT (median, range)**	3 (1-4)	3 (1-4)	4 (1-4)	0.674
**CVC**				0.019
Yes	27 (56.3)	20 (48.8)	7 (100.0)	
No	18 (37.5)	18 (46.2)	0 (0.0)	
*Unknown*	3 (6.3)	3 (7.3)	0 (0.0)	
**Khorana Score**				0.643
Low risk (1-2)	39 (81.3)	32 (78.1)	7 (100.0)	
High risk (≥3)	3 (6.3)	3 (7.3)	0 (0.0)	
*Missing*	6 (12.5)	6 (14.6)	0 (0.0)	
**ONKOTEV Score**				0.603
Low risk (0-1)	36 (75.0)	29 (70.8)	7 (100.0)	
High risk (≥2)	6 (12.5)	6 (14.6)	0 (0.0)	
*Missing*	6 (12.5)	6 (14.6)	0 (0.0)	

*p-value < .05, indicating statistically significant differences.

^†^ Two patients had recurrent disease with higher tumoral markers without measurable disease.

^‡^ Staging according to the American Joint Committee on Cancer (AJCC) 8th edition

^§^ One patient treated with Carboplatin and gemcitabine, and other with TIP (Paclitaxel, Ifosfamide and Cisplatin)

^¶^ One patient doesn’t receive treatment due to multiple organ dysfunction with refractory shock.

BEP, Bleomycin, Etoposide and Cisplatin; ChT, Chemotherapy; CR, Complete Response; CVC, Central Venous Catheter; EP, Etoposide and Cisplatin; IGCCCG, International Germ Cell Cancer Collaborative Group LN, Lymph Nodes; PD, Progression Disease; PR, Partial Response; SD, Stable Disease; TE, Thromboembolism; TGCT, Testicular Germ Cell Tumor.

#### 3.2.1 Thromboembolic events

TE events occurred in seven of the 48 patients in the advanced/recurrent cohort (14.6%). Notably, six events (85.7%) were diagnosed within 30 days of chemotherapy initiation, and all occurred during first-line treatment, except for one detected during second-line TIP. No event was observed during HDCT with ASCT. All TE cases occurred in patients with a CVC, though only two (28.6%) were directly classified as catheter-associated thrombosis. No TE event occurred in patients with stage I disease.

Metastatic pattern was significantly associated with TE (*p* = 0.021). Non−pulmonary visceral metastases (4, 57.1% vs. 4, 9.8%; *p* = 0.005), and lung metastases (1, 14.3% vs. 4, 9.8%; *p* = 0.020) were more common in the TE group. Conversely, LN−only disease was more frequent in the non−TE group (2, 28.6% vs. 31, 75.6% ; *p* = 0.174). While advanced staging showed a trend toward higher TE incidence in stage III (5, 71.4% vs. 12, 29.2%; *p* = 0.074), IGCCCG prognostic category emerged as the most significant clinical predictor for TE events, with poor-risk patients showing a significantly higher incidence compared to good or intermediate-risk patients (4, 57.1% vs. 6, 14.6%; *p* = 0.003).

Treatment-related factors, histology, response rates, and TE-risk scores did not differ significantly between the groups (**Table 2**). TE patients received a median of four chemotherapy cycles versus three in non−TE patients (*p* = 0.674) and achieved fewer complete responses (TE *n* = 1, 14.3% vs. non-TE *n* = 11, 26.8%; *p* = 0.526). Most notably, standard assessment tools failed to show predictive value in this population: although the Khorana and ONKOTEV scores identified a subset of high-risk patients (6.3% and 12.5%, respectively), none of the patients who experienced a TE event was classified as high-risk by either scale (*p* = 0.643 and *p* = 0.603, respectively).

Regarding the nature of the events, five (71.4%) were diagnosed in the outpatient setting. Arterial TE was observed in two (28.6%) patients, venous TE in two (28.6%), and mixed events in three (42.9%). Pulmonary embolism was the most frequent manifestation (*n* = 4), occurring either in isolation (*n* = 2) or associated with limb DVT and/or IVC thrombosis (one with both and one associated with IVC thrombosis only).The remaining cases included two patients with isolated limb DVT and one with isolated IVC thrombosis. Four events (57.1%) were symptomatic, and one patient (14.3%) died within 30 days of TE diagnosis due to TE. All TE patients received LMWH treatment according to established guidelines. In a univariate binomial logistic regression model, no significant correlations were found between TE events and clinical and pathological characteristics.

#### 3.2.2 Survival

At the time of database lock (December 31, 2024), the median follow-up was 53.0 months (range, 0.4–221.7), 31.5 months in the TE group (range, 2.3–89.8), and 53.8 months in the non-TE group (range, 0.4–221.7).

Of the 48 patients with advanced/recurrent disease, 41 (85.4%) remain alive: five (71.4%) in the TE-group and 36 (87.8%) in the non-TE group. Only one death was directly attributed to a TE event. Three patients (all IGCCCG poor prognostic group) died from disease progression 12 days, 2 months, and 33.4 months after diagnosis, respectively. Two further deaths were associated with chemotherapy toxicity, specifically one from febrile neutropenia with septic shock and another from ARDS (acute respiratory distress syndrome), potentially linked to bleomycin treatment, though this was not definitively confirmed. For one patient, the cause of death could not be determined.

**Figure 2:**
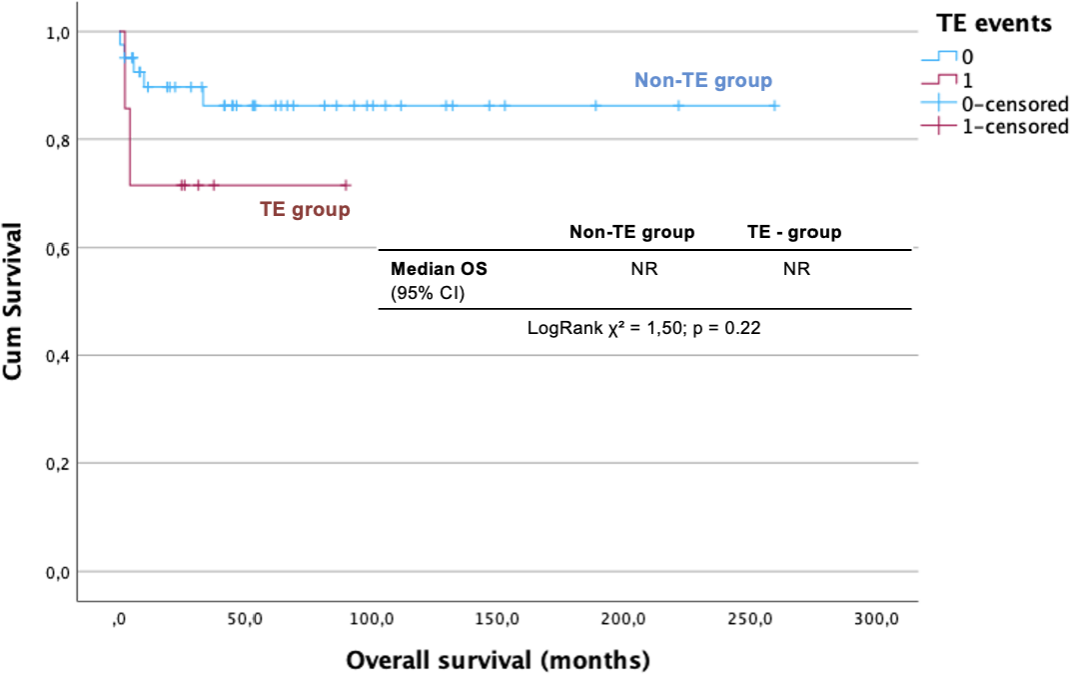
OS analysis for advanced-stage TGCT according to TE occurrence. CI, Confidence Interval; TE, Thromboembolism; OS, Overall survival.

The median OS was not reached, with no difference between TE and non-TE groups (log-rank *χ*² = 1.50, *p* = 0.22). The 5-year OS was 83.9% (standard deviation, 78.2%–89.6%), 86.2% (SD, 80.4%–92.0%) in the non-TE group and 71.4% (SD, 54.3%–88.5%) in the TE group (**Figure 2**).

**Figure 3:**
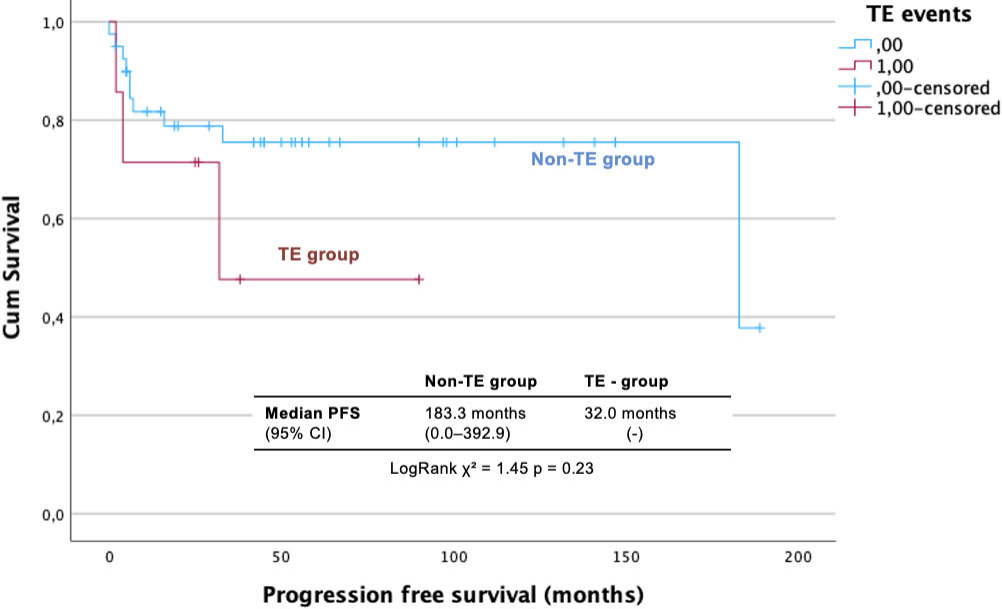
Progression free survival for advanced stage TGCT according to TE occurrence. CI, Confidence Interval; DFS, Disease Free Survival; TE, Thromboem-Bolism.

Regarding PFS, 15 patients (31.3%) experienced disease relapse, including eight in the favorable-risk group and six in the poor-risk IGCCCG group (one not classified). Recurrence rates were more frequent in the TE group [three (42.8%) in the TE group vs. 12 (29.3%) in the non-TE group]. Although the median PFS was numerically longer in non-TE patients, it is not statistically different [median PFS for the overall population: 183.0 months (95% CI, 0.0–392.9); 32.0 months (95% CI, not estimable) in the TE group vs. 183.0 months (95% CI, 0.0–392.9) in the non-TE group; log-rank *χ*² = 1.45, *p* = 0.23] (**Figure 3**). The 5-year PFS was 72.0% (SD, 65.0%–79.0%) for the overall group, 47.6% (25.1%–70.1%) in the TE group, and 75.5% (68.3%–82.7%) in the non-TE group. Almost all events occurred within the first 3 years of follow-up (*n* = 14, 83.3%). A single late recurrence was documented 15 years after initial treatment in a patient with a mixed germ-cell tumor containing a teratocarcinoma component. The relapse presented as mature teratoma involving the lungs and retroperitoneal lymph nodes and was treated with four cycles of BEP, followed by retroperitoneal lymph node dissection of the residual mass, which confirmed the teratoma.

## 4 Discussion

This retrospective multicenter study evaluated the incidence, risk factors, and prognostic impact of TE events in 136 patients with TGCT treated across three Portuguese oncology centers. The overall incidence of TE was 5.1%, with all events occurring in patients with advanced disease, corresponding to 15.2% within this subgroup. No TE events were reported in stage I patients, including those who received adjuvant chemotherapy. These findings are consistent with previously reported TE rates of approximately 10% in advanced TGCT populations, with variation depending on disease stage, treatment intensity, and baseline risk factor distribution (6–9).

The association between disease burden and TE risk was reinforced as a primary driver of these events (7–9). In our cohort, the IGCCCG prognostic category emerged as the most significant clinical predictor for TE events. Poor-risk patients showed a significantly higher incidence of TE compared to good or intermediate-risk patients (57.1% vs. 14.6%; *p* = 0.003). This was further supported by the significant association with metastatic patterns, where non-pulmonary visceral metastases (*p* = 0.005) and lung metastases (*p* = 0.020) were more common in the TE group. Nevertheless, no correlations were found between clinicopathological features and TE occurrence in logistic regression.

All TE cases occurred in patients with CVC, although only a minority were directly attributed to catheter thrombosis, suggesting that CVC use may be a surrogate of advanced disease or treatment intensity rather than an independent risk factor for TE. This observation may be influenced by biases, such as the small sample size and short chemotherapy duration in this population. The TE risk in patients with a CVC increases with treatment duration: it is low in the first month but rises significantly after 120 days of use (24). In TGCT patients, who typically receive only three to four cycles of platinum-based chemotherapy, this shorter exposure likely explains the low rate of catheter-related events compared to other cancers requiring prolonged regimens.

Furthermore, we analyzed patients receiving salvage therapy and HDCT, which require more complex and prolonged CVC placement, to determine whether higher treatment intensity in later lines influenced TE events. Despite this increased complexity, six of the seven TE events occurred during first-line treatment, one during second-line therapy, and not in the single patient who received third-line chemotherapy with HDCT. These findings reinforce the hypothesis that acute vascular toxicity from initial cisplatin exposure and primary bulky disease are the primary drivers of thrombosis in TGCT, surpassing the influence of subsequent treatment duration or intensity.

Unlike large cooperative studies reporting a significant negative impact of TE on survival (23), we did not observe statistically significant survival differences between TE and non-TE patients. The 5-year OS was 71.4% in TE and 86.2% in non-TE group (*p* = 0.22), while 5-years PFS was 47.6% in TE and 55.8% in non-TE group, with a median PFS of 31.5 and 29.3 months, respectively (*p* = 0.74). This discrepancy may reflect the limited number of events, which reduced the statistical power, as well as improvements in supportive care. Importantly, one death was directly attributed to TE, highlighting its clinical relevance even in a highly curable malignancy.

The timing and pattern of TE events were also consistent with prior reports, with most occurring within 30 days of chemotherapy initiation and pulmonary embolism being the most frequent manifestation. The early clustering of TE events reinforces the importance of vigilance during the first cycles of chemotherapy, when cisplatin-driven endothelial damage and inflammatory cytokines peak (6–8, 10–13). This period coincides with maximum biological stress, including direct vascular apoptosis and pro-coagulant factor release (14, 15).

Crucially, our study reveals a significant gap in current TE risk assessment tools. While the Khorana and ONKOTEV scores are validated across various malignancies, these scores failed to identify any of the patients who developed TE in our cohort, where 100% (7/7) of TE events occurred in patients classified as “low-risk” by Khorana (*p* = 0.643) and ONKOTEV (*p* = 0.603) scores. This suggests that in TGCT, the pro-thrombotic drivers are not the systemic factors measured by these scores (such as BMI or leukocytosis) but rather disease-specific factors like high tumor burden and the acute endothelial toxicity of cisplatin-based regimens. Consequently, we hypothesize that the IGCCCG risk status—which was highly predictive in our study (*p* = 0.003)—should be considered a more reliable indicator than traditional VTE scores when deciding on clinical monitoring or the need for thromboprophylaxis.

This study provides one of the few real-world, multicenter analyses of TE in TGCT within a European and specifically Portuguese population, offering region-specific insights to support clinical decision-making. The availability of detailed clinical annotation and standardized methodology enabled a meaningful assessment of risk factors despite the small number of events.

Clinically, our findings support current guideline recommendations to avoid CVC use whenever feasible (16, 17), highlight the limitations of cancer global thromboembolic risk scores, and suggest that routine prophylaxis is likely unnecessary in stage I disease. In contrast, patients with advanced disease, poor IGCCCG prognosis, visceral metastases, or requiring CVCs represent a higher-risk subgroup that may benefit from closer monitoring and potentially prophylactic strategies.

Several limitations must be acknowledged. The retrospective design is subject to selection and information bias, and the small number of TE events (*n* = 7) limited the statistical power to conduct multivariable analyses. TE incidence may also have been underestimated, as asymptomatic events were unlikely to be detected in routine clinical practice.

In conclusion, in this cohort of patients with TGCT, TE events occurred exclusively in patients with advanced disease and were strongly associated with tumor burden and central venous catheter use. Importantly, existing risk scores (Khorana and ONKOTEV) failed to predict any events, whereas the IGCCCG poor-risk category emerged as a highly significant predictor. While there were no statistically significant differences in survival between TE and non-TE patients, the recurrence rates were higher and the PFS was numerically shorter in the TE group, supporting its clinical relevance even in a highly curable malignancy. The less pronounced prognostic impact compared with previous large studies likely reflects the limited number of events and improvements in supportive care. Prospective studies are warranted to validate TGCT-specific risk stratification tools and to determine whether risk-adapted thromboprophylaxis can safely reduce TE incidence and improve outcomes in high-risk patients.

## Data Availability

The data analyzed in this study is subject to the following licenses/restrictions: Pseudo-anonimization, dataset from EMR, only shared among investigators. Requests to access these datasets should be directed to joanadalbuquerque@gamil.com.
